# Cultural and systemic determinants of medication adherence management: mixed-methods evidence from Jordanian healthcare professionals

**DOI:** 10.3389/fpubh.2025.1707561

**Published:** 2026-01-05

**Authors:** Anas Abed, Mohammad Abu Assab, Zainab Zakaraya, Wael Abu Dayyih, Badriyah S. Alotaibi, Nawal Alsubaie

**Affiliations:** 1 Department of Biopharmaceutics and Clinical Pharmacy, Faculty of Pharmacy, Al-Ahliyya Amman University, Amman, Jordan; 2 Clinical Pharmacy Department, Faculty of Pharmacy, Zarqa University, Zarqa, Jordan; 3 Faculty of Pharmacy, Mutah University, Alkarak, Jordan; 4 Department of Pharmaceutical Sciences, College of Pharmacy, Princess Nourah bint Abdulrahman University, Riyadh, Saudi Arabia; 5 Department of Pharmacy Practice, College of Pharmacy, Princess Nourah bint Abdulrahman University, Riyadh, Saudi Arabia

**Keywords:** adherence barriers, cultural facilitators, healthcare professionals, Jordan, medication adherence, mixed-methods, multimorbidity, pharmacists

## Abstract

**Background:**

Medication adherence is a critical determinant of therapeutic outcomes in chronic disease management, particularly for patients with multimorbidity. In Jordan, little is known about how healthcare professionals (HCPs) promote, assess, and address adherence, or how cultural factors influence their practices. This study examined adherence management strategies among pharmacists and physicians, integrating quantitative and qualitative evidence to identify barriers, facilitators, and training needs.

**Methods:**

A mixed-methods, cross-sectional study was conducted with 390 HCPs (273 pharmacists, 117 physicians) recruited via convenience sampling from community and hospital settings across Jordan. Participants completed a structured, self-administered survey assessing demographic/practice characteristics, adherence promotion strategies, assessment methods, barriers, facilitators, and collaboration practices. The survey instrument was a structured questionnaire developed from prior adherence literature and refined through pilot testing. A purposive subset of 20 survey participants (12 pharmacists, 8 physicians) participated in semi-structured interviews to explore these themes in depth. The quantitative phase used convenience sampling across healthcare settings in Jordan, while qualitative participants were purposively selected from the survey pool to ensure diversity in profession and practice setting Quantitative data were analyzed using descriptive statistics, chi-square tests, t-tests, and multivariable logistic regression. Qualitative data were analyzed inductively using Braun and Clarke’s thematic analysis. A convergent mixed-methods design was employed, integrating quantitative findings with qualitative themes during interpretation.

**Results:**

Pharmacists more frequently provided patient education (88.3% vs. 71.8%, *p* < 0.001) and simplified regimens (65.2% vs. 47.9%, *p* = 0.001) than physicians. Structured tools (pill counts, electronic monitoring) were infrequently used, while less structured methods such as self-reports and refill data were more common. Common barriers included time constraints (68.0%), low health literacy (54.7%), and medication costs (48.7%). Cultural facilitators such as prayer-based reminders (38.0%) and family support (42.3%) were reported by over one-third of participants, particularly in community practice. Only 18.2% had received formal adherence training, which predicted greater use of structured interventions. Qualitative insights highlighted systemic resource limitations, the value of trust-based counseling, and the need for stronger interprofessional collaboration.

**Conclusion:**

Pharmacists play a leading role in adherence support in Jordan, leveraging patient education, regimen simplification, and culturally anchored strategies. However, both pharmacists and physicians face systemic and patient-level barriers, with limited training and collaboration hindering impact. Expanding adherence training, formalizing interprofessional roles, and incorporating cultural facilitators into routine care could strengthen adherence management for multimorbid patients in Jordan and similar contexts.

## 1 Introduction

Medication adherence is a cornerstone of effective chronic disease management and a critical determinant of patient outcomes (1). Poor adherence is associated with increased morbidity, mortality, and healthcare costs, particularly among patients with multimorbidity who often require complex medication regimens (2). Globally, the World Health Organization estimates that only about half of patients with chronic conditions adhere to prescribed therapies, with adherence rates in low- and middle-income countries frequently lower due to systemic, economic, and cultural factors (3, 4). In the Middle East, including Jordan, multimorbidity is increasingly prevalent, further amplifying the need for effective adherence management strategies, particularly given the high prevalence of polypharmacy and medication-related problems in chronic disease patients (5).

Healthcare professionals (HCPs), particularly pharmacists and physicians, play a pivotal role in supporting adherence through patient education, regimen simplification, monitoring, and tailored interventions (6, 7). However, these efforts are often hindered by barriers such as time constraints, low patient health literacy, medication costs, and fragmented care systems (8). At the same time, cultural factors, including religious practices and family support, can also influence adherence behaviors (9). While international literature highlights the importance of integrating cultural and contextual considerations into adherence promotion, there is limited empirical evidence from Jordan exploring how pharmacists and physicians approach this in practice. Moreover, no previous study in Jordan has employed a mixed-methods design to simultaneously examine quantifiable adherence-support practices and the professional reasoning that underpins them.

Previous research in the region has largely focused on patient-reported adherence or disease-specific adherence interventions, with relatively few studies comparing HCP practices across professions or integrating both quantitative and qualitative perspectives. Furthermore, little is known about the extent to which cultural facilitators are utilized, the barriers encountered in practice, and the role of formal adherence training in shaping professional behavior. To address these limitations, a mixed-methods design was chosen to quantify current adherence-support practices while also exploring the contextual, experiential, and system-level factors that cannot be fully captured through survey data alone.

This study addresses these gaps by investigating medication adherence management practices among pharmacists and physicians in Jordan using a mixed-methods approach. The quantitative component measures the prevalence of adherence promotion strategies, assessment methods, barriers, facilitators, and collaboration practices, while the qualitative component explores the systemic, professional, cultural, and patient-level factors influencing these practices. By integrating these findings, the study aims to provide a comprehensive understanding of adherence management in the Jordanian healthcare context and to identify opportunities for enhancing training, collaboration, and culturally sensitive care. The development of the survey items was informed by the WHO Five Dimensions of Adherence framework, which categorizes adherence barriers into patient-related, condition-related, therapy-related, health-system, and socioeconomic dimensions (10).

## 2 Methodology

### 2.1 Study design

This study employed a mixed-methods, cross-sectional design to investigate medication adherence management practices among healthcare professionals (HCPs) in Jordan, focusing on pharmacists and physicians. This convergent mixed-methods design was selected because medication adherence is influenced by both measurable practice patterns and contextual professional reasoning. Integrating quantitative and qualitative findings allowed us to capture not only the frequency of adherence-support activities but also the underlying factors shaping clinicians’ decisions. The quantitative component utilized a structured survey to collect data on demographic characteristics, adherence promotion strategies, assessment methods, barriers, facilitators, and interdisciplinary collaboration. The qualitative component involved semi-structured interviews to provide deeper insights into systemic, professional, cultural, and patient-related factors influencing adherence management, particularly for patients with multimorbidity.

### 2.2 Participants and sampling

#### 2.2.1 Quantitative survey

A convenience sampling strategy was used to recruit 390 HCPs (273 pharmacists, 70.0%; 117 physicians, 30.0%) from community and hospital settings across Jordan. The numerical imbalance between pharmacists and physicians reflects the actual professional distribution in Jordan, where pharmacists constitute a substantially larger proportion of the medication-management workforce and are more accessible in community and hospital settings. Because the study employed convenience sampling, the higher number of pharmacists reflects natural response patterns rather than intentional oversampling. The achieved sample sizes for both groups remained adequate for the planned comparative analyses, as statistical power calculations were based on expected between-group differences rather than equal group sizes. The target sample size was calculated to achieve 80% power to detect an expected absolute difference of 15% in the prevalence of adherence-support strategy use between pharmacists and physicians, with a two-sided *α* = 0.05. This difference was chosen as a conservative estimate based on regional literature indicating that pharmacists generally report higher engagement in adherence-promotion activities than physicians, although precise numerical estimates of this difference are not consistently reported in Middle Eastern studies (11, 12).

Inclusion criteria were: (1) licensed pharmacists or physicians practicing in Jordan, (2) at least 1 year of professional experience, and (3) involvement in medication adherence management for patients with chronic conditions. Exclusion criteria included non-practicing HCPs or those in administrative roles.

Participants were invited to take part in the survey through multiple channels, including professional networks such as the Jordan Pharmacists Association, as well as outreach to community pharmacies, outpatient clinics, and public/private hospitals. The invitation included detailed information about the study and a link to the online questionnaire hosted on Qualtrics. The survey link was distributed electronically via email, WhatsApp, and Telegram, and also through professional groups and social media platforms. Data collection took place over a 3-month period (October–December 2024), with follow-up reminders sent electronically and via phone to encourage participation.

#### 2.2.2 Qualitative interviews

A subset of 20 HCPs (12 pharmacists, 8 physicians) was purposively selected from those who completed the quantitative survey sample for semi-structured interviews to explore adherence management practices in depth. Selection criteria included diversity in profession, practice setting (community vs. hospital), region, and adherence training status to capture varied perspectives. The sample size (*n* = 20) was determined based on thematic saturation, confirmed when no new themes emerged after 18 interviews, with two additional interviews to ensure data robustness. Participants were contacted via email, with a 90% acceptance rate (20/22 invited).

### 2.3 Data collection

#### 2.3.1 Quantitative survey

A structured, self-administered survey was developed from a literature review of validated adherence-support instruments used in previous research on healthcare professionals (13–17), ensuring that the survey reflected established behavioral, practical, and systemic determinants of medication adherence.

To establish content validity, the draft questionnaire was reviewed by a panel of five experts (three clinical pharmacy academics and two practicing physicians) who assessed the relevance, clarity, and cultural suitability of each item. Following expert feedback, minor linguistic and contextual adjustments were made. The survey comprised six domains: (1) demographic and practice characteristics, (2) adherence promotion strategies, (3) adherence assessment methods, (4) barriers to adherence management, (5) cultural facilitators, and (6) relationship-based facilitators and polypharmacy.

Items were rated on a 5-point Likert scale (1 = Never to 5 = Always), with “frequent” defined as ≥4. The instrument was piloted with 30 HCPs (20 pharmacists, 10 physicians) for clarity and cultural appropriateness. Internal consistency was evaluated during the pilot phase (*n* = 30), with Cronbach’s alpha of 0.87. The final survey, administered in Arabic via Qualtrics, required approximately 10–15 min to complete.

#### 2.3.2 Qualitative interviews

A purposive subset of survey respondents (*n* = 20; 12 pharmacists, 8 physicians) was invited for semi-structured interviews to explore survey-identified patterns in depth. Selection ensured variation in profession, practice setting, region, and adherence training status.

The interview guide was developed using two sources: (1) emergent findings from the quantitative survey and (2) the WHO Five Dimensions of Adherence framework, which conceptualizes adherence as influenced by patient-related, condition-related, therapy-related, health-system, and socioeconomic factors. This framework ensured that the guide systematically captured behavioral, contextual, structural, and cultural determinants of adherence-support practices. It shaped the formulation of open-ended questions and the follow-up probes used to explore participants’ reasoning and lived experiences.

Core open-ended questions included:

- “Can you describe how you approach medication adherence with patients who have multiple chronic conditions?”
- “What strategies do you use to help patients remember their medications?”
- “How do you address adherence in patients with low literacy?”
- “What role do cultural or religious practices (e.g., prayer times) play in supporting adherence?”
- “What challenges do you face in collaborating with other healthcare professionals regarding adherence management?”
- “How has any training you have received affected your adherence management practices?”

Interviews, conducted in Arabic by a trained researcher, lasted 30–45 min and took place in-person face to face. Sessions were audio-recorded with consent, transcribed verbatim in Arabic, and translated into English by a bilingual researcher, with translations cross-checked by a second translator for accuracy.

Follow-up probes were used to encourage elaboration and capture detailed examples. Thematic saturation was monitored throughout data collection. Emerging codes were reviewed after each interview, and by the 18th interview no new themes were identified. Two additional interviews were conducted to confirm saturation, giving a final total of 20 interviews. This decision was based on consensus between the two primary coders and was verified by a third researcher.

### 2.4 Data analysis

Quantitative data were analyzed using Prism 9.0 version software. Descriptive statistics (means, standard deviations, frequencies, and percentages) summarized demographic and practice characteristics, adherence strategies, assessment methods, barriers, facilitators, and polypharmacy/collaboration. Independent t-tests compared continuous variables between pharmacists and physicians, while chi-square tests compared categorical variables. Multivariable logistic regression identified predictors of frequent practice (Likert ≥ 4) for adherence strategies, assessment methods, facilitators, and reporting of barriers or polypharmacy challenges, adjusting for age, gender, years of experience, practice setting, and adherence training.

Prior to performing logistic regression, key statistical assumptions were evaluated. Multicollinearity was assessed using variance inflation factors (VIF), with all predictors showing VIF values below 2.0, indicating no problematic multicollinearity. Model fit was assessed using the Hosmer–Lemeshow goodness-of-fit test (*p* > 0.05 for all models) and Nagelkerke’s pseudo-R^2^ to evaluate explanatory power. Cases with missing data (< 5% of total responses) were excluded from regression analyses using listwise deletion; missingness was examined and determined to be random, and its exclusion did not materially affect the results. Odds ratios (ORs) and 95% confidence intervals (CIs) were reported, with statistical significance set at *p* < 0.05.

Qualitative data were analyzed inductively using Braun and Clarke’s (2006) thematic analysis framework in NVivo (version 12). Two coders independently reviewed transcripts for familiarization and generated initial codes (e.g., “high patient volumes,” “prayer-based reminders”). Codes were grouped and refined into coherent themes, resulting in 16 final themes. Themes were reviewed against transcripts for consistency and distinctiveness. A third researcher independently reviewed 50% of coded transcripts, achieving 90% interrater agreement—considered high for qualitative research—with discrepancies resolved through discussion. Analysis was conducted in Arabic to preserve cultural nuances, with translations into English cross-checked for accuracy. Themes were mapped to systemic, professional, cultural, and patient-related domains and linked to relevant quantitative findings to facilitate mixed-methods integration.

Integration of the quantitative and qualitative strands occurred at the interpretation stage, following a convergent mixed-methods approach. Quantitative data identified the prevalence and patterns of adherence-related practices, barriers, and facilitators, while qualitative themes contextualized these patterns and explained underlying reasons and systemic influences.

### 2.5 Ethical considerations

The study received ethical approval from the Institutional Review Board of Al-Ahliyya Amman University (IRB No. AAU/2/2/2024–2025). All participants were informed about the purpose of the study and procedures and provided written informed consent prior to participation. Participation was voluntary, and anonymity was safeguarded by assigning unique identifiers to each respondent.

## 3 Results

### 3.1 Demographic and practice characteristics of study participants

A total of 390 healthcare professionals (HCPs) completed the survey, with 273 pharmacists (70.0%) and 117 physicians (30.0%). The mean age was 34.2 years (SD = 7.8), with no significant difference between professions. Most had 5–15 years of experience (62.3%), and practice settings varied: 48.7% worked in community pharmacies, 15.9% in outpatient clinics, 21.3% in public hospitals, and 14.1% in private hospitals. Pharmacists were concentrated in community pharmacies (69.6%), while physicians were distributed across hospital and clinic settings.

Formal training in medication adherence counseling was reported by 18.2% of participants, more common among pharmacists (22.7%) than physicians (7.7%, *p* < 0.01). Consultation patterns differed significantly where pharmacists were more likely to spend 5–10 min with patients (49.8% vs. 32.5%, *p* < 0.01), while hospital physicians frequently spent less than 5 min (41.0%). Despite longer consultation times, pharmacists still reported higher time-constraint pressures, reflecting high daily patient volumes in community settings (Table 1).

**Table 1:** Demographic and practice characteristics of healthcare professionals (self-reported survey responses; *N* = 390).

Characteristic	Total (*N* = 390)	Pharmacists (*n* = 273)	Physicians (*n* = 117)	*p*-value
Age, years (Mean ± SD)	34.2 ± 7.8	33.8 ± 7.5	35.1 ± 8.3	0.16
Gender, n (%)				0.17
Male	228 (58.5)	153 (56.0)	75 (64.1)	
Female	162 (41.5)	120 (44.0)	42 (35.9)	
Years of Experience, n (%)				0.39
<5 years	88 (22.6)	65 (23.8)	23 (19.7)	
5–15 years	243 (62.3)	171 (62.6)	72 (61.5)	
>15 years	59 (15.1)	37 (13.6)	22 (18.8)	
Practice Setting, n (%)				<0.001
Community Pharmacy	190 (48.7)	190 (69.6)	0 (0.0)	
Outpatient Clinic	62 (15.9)	30 (11.0)	32 (27.4)	
Public Hospital	83 (21.3)	40 (14.7)	43 (36.8)	
Private Hospital	55 (14.1)	13 (4.8)	42 (35.9)	
Region, n (%)				0.95
Central (e.g., Amman, Zarqa)	164 (42.1)	114 (41.8)	50 (42.7)	
North (e.g., Irbid, Mafraq)	121 (31.0)	86 (31.5)	35 (29.9)	
South (e.g., Aqaba, Karak)	105 (26.9)	73 (26.7)	32 (27.4)	
Education Level, n (%)				0.5
Bachelor’s (BPharm)	249 (63.8)	179 (65.4)	70 (59.8)	
PharmD/Specialty Certification	114 (29.2)	77 (28.2)	37 (31.6)	
Master’s or Higher	27 (6.9)	17 (6.4)	10 (8.5)	
Adherence Training, n (%)				<0.01
Yes	71 (18.2)	62 (22.7)	9 (7.7)	
No	319 (81.8)	211 (77.3)	108 (92.3)	
Time Spent Talking to Patients About Medication Use, n (%)				<0.01
<5 min	124 (31.8)	76 (27.8)	48 (41.0)	
5–10 min	174 (44.6)	136 (49.8)	38 (32.5)	
10–15 min	72 (18.5)	48 (17.6)	24 (20.5)	
>15 min	20 (5.1)	13 (4.8)	7 (6.0)	

All practice-related variables, including estimated time spent on patient counseling, were self-reported by participants.

### 3.2 High prevalence of patient education and simplified regimens, with pharmacists leading in counseling

Patient education (82.7%) and simplified regimens (60.0%) were the most frequently reported adherence-support strategies. Pharmacists reported higher use of both education (88.3% vs. 71.8%, *p* < 0.001) and simplification (65.2% vs. 47.9%, *p* = 0.002) compared to physicians. Reminder systems were used by 45.3% of HCPs, with no significant overall difference, though more common among community pharmacists than public-hospital staff (50.0% vs. 35.7%, *p* = 0.02). Regression analysis confirmed that being a pharmacist and having adherence training were associated with higher odds of providing education and regimen simplification (Table 2).

**Table 2:** Prevalence of adherence promotion strategies by profession (self-reported survey items; *N* = 390).

Strategy	Total (*N* = 390)	Pharmacists (*n* = 273)	Physicians (*n* = 117)	*p*-value	OR (95% CI)
Patient Education, n (%)				<0.001	3.22 (1.94–5.36)
Frequent (Likert ≥4)	323 (82.7)	241 (88.3)	82 (71.8)		
Infrequent (Likert <4)	67 (17.3)	32 (11.7)	35 (28.2)		
Simplified Regimens, n (%)				0.002	2.04 (1.31–3.17)
Frequent (Likert ≥4)	234 (60.0)	178 (65.2)	56 (47.9)		
Infrequent (Likert <4)	156 (40.0)	95 (34.8)	61 (52.1)		
Reminder Systems, n (%)				0.42	
Frequent (Likert ≥4)	177 (45.3)	128 (46.9)	49 (41.9)		1.23 (0.77–1.96)
Infrequent (Likert <4)	213 (54.7)	145 (53.1)	68 (58.1)		

*p-values from chi-square tests. Frequent use defined as Likert score ≥4 (Often or Always) on a 5-point scale (1 = Never, 5 = Always).

### 3.3 Limited use of structured adherence assessment tools, with reliance on self-reports and refill data

Structured, objective adherence assessment tools—such as pill counts and electronic monitoring—were rarely used, reported by only 28.2 and 12.6% of participants, respectively. In contrast, less structured methods were more common: patient self-reports were used frequently by 76.4% of participants, pharmacy refill data by 62.3%, and treatment response evaluation by 56.9%. Pharmacists reported significantly more frequent use of pharmacy refill data compared to physicians (68.5% vs. 47.9%, *p* < 0.001), whereas physicians reported more frequent use of treatment response evaluation (68.4% vs. 52.0%, *p* = 0.002; Table 3).

**Table 3:** Prevalence of adherence assessment methods by profession (self-reported survey items; *N* = 390).

Assessment method	Total (*N* = 390)	Pharmacists (*n* = 273)	Physicians (*n* = 117)	*p*-value	OR (95% CI)
Patient Self-Reports, n (%)				0.2	1.42 (0.87–2.32)
Frequent (Likert ≥4)	298 (76.4)	214 (78.4)	84 (71.8)		
Infrequent (Likert <4)	92 (23.6)	59 (21.6)	33 (28.2)		
Pharmacy Refill Data, n (%)				<0.001	2.36 (1.48–3.76)
Frequent (Likert ≥4)	243 (62.3)	187 (68.5)	56 (47.9)		
Infrequent (Likert <4)	147 (37.7)	86 (31.5)	61 (52.1)		
Pill Counts, n (%)				0.54	1.20 (0.72–2.01)
Frequent (Likert ≥4)	110 (28.2)	80 (29.3)	30 (25.6)		
Infrequent (Likert <4)	280 (71.8)	193 (70.7)	87 (74.4)		
Electronic Monitoring, n (%)				0.67	0.87 (0.44–1.72)
Frequent (Likert ≥4)	49 (12.6)	33 (12.1)	16 (13.7)		
Infrequent (Likert <4)	341 (87.4)	240 (87.9)	101 (86.3)		
Treatment Response Evaluation, n (%)				0.004	0.50 (0.32–0.79)
Frequent (Likert ≥4)	222 (56.9)	142 (52.0)	80 (68.4)		
Infrequent (Likert <4)	168 (43.1)	131 (48.0)	37 (31.6)		

Frequent use defined as Likert score ≥4 (Often or Always) on a 5-point scale (1 = Never, 5 = Always).

### 3.4 Time constraints and medication costs as major barriers to adherence management

Time constraints were the most reported barrier (68.0%), followed by low patient health literacy (54.7%) and medication costs (48.7%). Pharmacists more frequently cited time pressure (72.5% vs. 57.3%, *p* = 0.004), while physicians more often reported cost as a barrier (57.3% vs. 45.1%, *p* = 0.036). Low health literacy affected both professions similarly. Analyses suggested that pharmacist profession was associated with higher odds of reporting time constraints (OR = 2.0, 95% CI: 1.25–3.10), while being a physician was associated with higher odds of citing medication cost (OR = 1.6, 95% CI: 1.05–2.50; Table 4).

**Table 4:** Prevalence of barriers to adherence management by profession (self-reported survey items; *N* = 390).

Barrier	Total (*N* = 390)	Pharmacists (*n* = 273)	Physicians (*n* = 117)	*p*-value	OR (95% CI)
Time Constraints, n (%)				<0.004	1.98 (1.25–3.10)
Frequent (Likert ≥4)	265 (68.0)	198 (72.5)	67 (57.3)		
Infrequent (Likert <4)	125 (32.0)	75 (27.5)	50 (42.7)		
Low Patient Health Literacy, n (%)				0.65	1.10 (0.71–1.70)
Frequent (Likert ≥4)	213 (54.7)	151 (55.3)	62 (53.0)		
Infrequent (Likert <4)	177 (45.3)	122 (44.7)	55 (47.0)		
Medication Costs, n (%)				0.036	0.61 (0.40–0.95)
Frequent (Likert ≥4)	190 (48.7)	123 (45.1)	67 (57.3)		
Infrequent (Likert <4)	200 (51.3)	150 (54.9)	50 (42.7)		
Inadequate Tracking Systems, n (%)				0.51	0.84 (0.54–1.31)
Frequent (Likert ≥4)	142 (36.4)	96 (35.2)	46 (39.3)		
Infrequent (Likert <4)	248 (63.6)	177 (64.8)	71 (60.7)		
Lack of Collaboration, n (%)				0.4	0.80 (0.50–1.27)
Frequent (Likert ≥4)	120 (30.8)	80 (29.3)	40 (34.2)		
Infrequent (Likert <4)	270 (69.2)	193 (70.7)	77 (65.8)		

*p-*values from chi-square tests. Frequent barriers defined as Likert score ≥4 (Often or Always) on a 5-point scale (1 = Never, 5 = Always). OR for pharmacists vs. physicians (pharmacists as reference).

### 3.5 Cultural facilitators, including prayer-based reminders, reported by over one-third of participants

As shown in Table 5, prayer-based reminders were reported by 38.0% of healthcare professionals, with similar rates across professions. In this study, prayer-based reminders referred to the use of daily prayer times as behavioral cues for medication taking. This strategy anchors medication use to an existing daily routine, helping patients maintain consistent dosing without relying on additional prompts.

**Table 5:** Prevalence of cultural facilitators by profession (self-reported survey items; *N* = 390).

Facilitator	Total (*N* = 390)	Pharmacists (*n* = 273)	Physicians (*n* = 117)	*p*-value	OR (95% CI)
Prayer-Based Reminders, n (%)				0.38	1.26 (0.80–1.98)
Frequent (Likert ≥4)	148 (38.0)	108 (39.6)	40 (34.2)		
Infrequent (Likert <4)	242 (62.0)	165 (60.4)	77 (65.8)		
Family Support, n (%)				0.042	1.63 (1.04–2.55)
Frequent (Likert ≥4)	165 (42.3)	125 (45.8)	40 (34.2)		
Infrequent (Likert <4)	225 (57.7)	148 (54.2)	77 (65.8)		
Religious Counseling, n (%)				0.53	0.83 (0.51–1.35)
Frequent (Likert ≥4)	100 (25.6)	67 (24.5)	33 (28.2)		
Infrequent (Likert <4)	290 (74.4)	206 (75.5)	84 (71.8)		

*p-*values from chi-square tests. Frequent use defined as Likert score ≥4 (Often or Always) on a 5-point scale (1 = Never, 5 = Always). OR for pharmacists vs. physicians (pharmacists as reference).

Family support, reported by 42.3% of participants, was more frequently reported by pharmacists than physicians (45.8% vs. 34.2%, *p* = 0.044), which may be associated with pharmacists’ engagement with patients and their families in community settings.

Religious counseling, reported by 25.6% of participants, differed from prayer-based reminders in that it involved motivational framing of adherence as part of a patient’s religious or spiritual duty. Examples included explaining that maintaining one’s health is a responsibility entrusted by God, or that taking prescribed medicines is part of caring for the body in accordance with faith.

### 3.6 Strong patient-provider relationships enhance adherence, especially in community pharmacies

Trust-based relationships were reported by 70.7% of HCPs, more common among pharmacists (75.5% vs. 59.8%, *p* = 0.003), especially in community pharmacies (78.4%). Personalized counseling was also more frequent among pharmacists (63.7% vs. 45.3%, *p* < 0.001). Follow-up communication was less common overall (35.1%) and did not differ significantly between professions (Table 6).

**Table 6:** Relationship-based facilitators and communication practices (self-reported survey items; *N* = 390).

Facilitator	Total (*N* = 390)	Pharmacists (*n* = 273)	Physicians (*n* = 117)	*p*-value	OR (95% CI)
Trust-Based Relationships, n (%)				0.003	2.06 (1.30–3.27)
Frequent (Likert ≥4)	276 (70.7)	206 (75.5)	70 (59.8)		
Infrequent (Likert <4)	114 (29.3)	67 (24.5)	47 (40.2)		
Personalized Counseling, n (%)				<0.001	2.12 (1.37–3.29)
Frequent (Likert ≥4)	227 (58.2)	174 (63.7)	53 (45.3)		
Infrequent (Likert <4)	163 (41.8)	99 (36.3)	64 (54.7)		
Follow-Up Communication, n (%)				0.54	1.18 (0.75–1.87)
Frequent (Likert ≥4)	137 (35.1)	99 (36.3)	38 (32.5)		
Infrequent (Likert <4)	253 (64.9)	174 (63.7)	79 (67.5)		

*p-*values from chi-square tests. Frequent use defined as Likert score ≥4 (Often or Always) on a 5-point scale (1 = Never, 5 = Always). OR for pharmacists vs. physicians (pharmacists as reference).

### 3.7 Polypharmacy challenges and interdisciplinary collaboration

Managing polypharmacy was challenging for 64.1% of HCPs, with similar prevalence across professions (65.2% of pharmacists vs. 61.5% of physicians; *p* = 0.56). Only 22.3% reported frequent interdisciplinary collaboration, and this did not differ significantly between pharmacists and physicians (21.6% vs. 23.9%; *p* = 0.71). These findings suggest that while polypharmacy remains a substantial challenge across practice settings, collaborative approaches to address it are still infrequent in routine care (Table 7).

**Table 7:** Collaborative practices and polypharmacy-related challenges (self-reported survey items; *N* = 390).

Characteristic	Total (*N* = 390)	Pharmacists (*n* = 273)	Physicians (*n* = 117)	*p*-value	OR (95% CI)
Difficulty Managing Polypharmacy, n (%)				0.56	1.17 (0.74–1.86)
Frequent (Likert ≥4)	250 (64.1)	178 (65.2)	72 (61.5)		
Infrequent (Likert <4)	140 (35.9)	95 (34.8)	45 (38.5)		
Interdisciplinary Collaboration, n (%)				0.71	0.87 (0.52–1.45)
Frequent (Likert ≥4)	87 (22.3)	59 (21.6)	28 (23.9)		
Infrequent (Likert <4)	303 (77.7)	214 (78.4)	89 (76.1)		

*p-*values from chi-square tests. Frequent use defined as Likert score ≥4 (Often or Always) on a 5-point scale (1 = Never, 5 = Always). OR for pharmacists vs. physicians (pharmacists as reference).

### 3.8 Training in adherence counseling predicts increased use of structured interventions

Training was associated with increased use of certain structured interventions. Specifically, trained healthcare professionals reported significantly higher use of reminder systems (57.7% vs. 38.2%, *p* < 0.01) and simplified regimens (71.8% vs. 54.2%, *p* = 0.01) compared to their untrained counterparts (Table 8). However, not all structured approaches demonstrated this pattern; other strategies, such as pill counts, electronic monitoring, or pharmacy refill data, were not assessed in relation to training in Table 8 and may not exhibit the same trend. Among trained professionals, pharmacists were more likely than physicians to employ reminder systems (62.9% vs. 22.2%, *p* = 0.03), while no significant difference was observed for simplified regimens.

**Table 8:** Predictors of frequent adherence-promotion practices (multivariable logistic regression model using self-reported survey variables; *N* = 390).

Intervention	Total (*N* = 390)	Trained (*n* = 71)	Untrained (*n* = 319)	Pharmacists trained (*n* = 62)	Physicians trained (*n* = 9)	*p*-value (trained vs. untrained)	OR (95% CI) – trained vs. untrained	*p*-value (pharmacists vs. physicians, trained)	OR (95% CI) – pharmacists vs. physicians (trained)
Reminder Systems, n (%)						<0.01	2.21 (1.31–3.72)	0.03	5.82 (1.10–30.86)
Frequent (Likert ≥4)	177 (45.4)	41 (57.7)	122 (38.2)	39 (62.9)	2 (22.2)				
Infrequent (Likert <4)	213 (54.6)	30 (42.3)	197 (61.8)	23 (37.1)	7 (77.8)				
Simplified Regimens, n (%)						0.01	2.16 (1.20–3.88)	0.26	2.17 (0.39–12.00)
Frequent (Likert ≥4)	234 (60.0)	51 (71.8)	173 (54.2)	46 (74.2)	5 (55.6)				
Infrequent (Likert <4)	156 (40.0)	20 (28.2)	146 (45.8)	16 (25.8)	4 (44.4)				

*p-*values are from chi-square tests. Frequent use defined as a Likert score ≥ 4 (Often or Always) on a 5-point scale (1 = Never, 5 = Always). OR compare trained vs. untrained healthcare professionals and trained physicians vs. trained pharmacists (pharmacists as the reference group).

### 3.9 Qualitative insights and linkage with the quantitative findings

The thematic analysis of 20 semi-structured interviews identified 16 key themes that enriched and contextualized the survey findings (Table 9). These themes reflected systemic, professional, cultural, and patient-related factors influencing medication adherence management in Jordan.

**Table 9:** Qualitative themes derived from semi-structured interviews (thematic analysis of 20 healthcare professionals).

Theme	Prevalence (% interviewees, *n* = 20)	Illustrative quote	Linked quantitative finding	Explanation
Systemic resource limitations	85% (*n* = 17)	“With the patient load we have, it’s impossible to give every case the time it deserves.” (Pharmacist)	Consultation time: 44.6% reported 5–10 min (Table 1); Time constraints: 68.0% (Table 4); Low use of electronic monitoring: 12.6% (Table 3)	High patient volumes and limited technology restrict consultation time and tool use, especially for physicians.
Community pharmacy interactions	65% (*n* = 13)	“Seeing regular customers lets me follow their progress closely over time.” (Pharmacist)	Pharmacists’ consultation time: 49.8% reported 5–10 min (Table 1); Trust-based relationships: 75.5% (Table 6)	Frequent visits in pharmacies enable longer consultations and rapport-building.
Limited training access	65% (*n* = 13)	“Workshops are rarely organized here, so our skills do not improve as fast as they should.” (pharmacist)	Adherence training: 18.2% overall (22.7% pharmacists vs. 7.7% physicians, *p* < 0.01); Reminder systems: 58.5% among trained respondents (Table 8)	Limited access to structured adherence counseling training is a widespread issue across Jordan
Tailored counseling	70% (*n* = 14)	“I adapt instructions for each patient, so they fit their personal routine.” (Pharmacist)	Patient education: 88.3% pharmacists (Table 2); Personalized counseling: 63.7% (Table 6)	Pharmacists’ counseling supports education and personalized approaches.
Prayer as a routine anchor	80% (*n* = 16)	“Linking medicine times with prayers helps patients remember without extra effort.” (Pharmacist)	Prayer-based reminders: 38.0% (Table 5); Simplified regimens: 60.0% (Table 2)	Aligning regimens with prayer times aids adherence for multimorbidity patients.
Cost barriers	70% (*n* = 14)	“Some patients ration their medicines to make them last longer.” (Physician)	Medication cost reported as a barrier: 57.3% physicians (Table 4)	Multimorbidity increases costs, noted more by physicians.
Low literacy challenges	65% (*n* = 13)	“When a prescription has many drugs, patients with low literacy get confused quickly.” (Pharmacist)	Low health literacy: 54.7% (Table 4); Unreliable self-reports: 76.4% (Table 3)	Low literacy complicates regimen adherence and self-report accuracy.
Unreliable self-reports	70% (*n* = 14)	“Patients often report taking their medicines even when they have missed several doses.” (Pharmacist)	Reliance on self-reports: 76.4% (Table 3)	Patients overreport adherence, driving reliance despite concerns.
Clinical outcomes focus	75% (*n* = 15)	“I rely on lab results to see if the treatment is actually working.” (Physician)	Treatment response monitoring: 68.4% physicians (Table 3)	Physicians prioritize clinical monitoring in hospitals.
Trust as a motivator	80% (*n* = 16)	“Patients open up about challenges when they know you have been following their case for years.” (Pharmacist)	Trust-based relationships: 75.5% pharmacists (Table 6)	Frequent pharmacist–patient interactions build trust, enhancing adherence.
Fragmented care	75% (*n* = 15)	“We rarely get updates from other providers, so changes can go unnoticed.” (Pharmacist)	Limited collaboration: 22.3% (Table 7); Inadequate tracking systems: 36.4% (Table 4)	Lack of shared records hinders polypharmacy management.
Need for multidisciplinary protocols	65% (*n* = 13)	“Joint planning meetings would make medication management much smoother.” (Pharmacist)	Limited collaboration: 22.3% (Table 7)	Calls for shared systems to improve collaboration.
Training gaps hinder practice	70% (*n* = 14)	“Without training, you stick to the simplest advice you know.” (Physician)	Only 18.2% reported training (22.7% pharmacists vs. 7.7% physicians, *p* < 0.01); Lower intervention use by untrained (38.4% reminders; 54.1% simplified regimens)	Untrained HCPs rely on basic methods.
Multimorbidity management	80% (*n* = 16)	“Coordinating five or more medicines for one patient is never easy.” (Pharmacist)	Polypharmacy challenges: 64.1% (Table 7); Simplified regimens: 60.0% (Table 2)	Complex regimens drive need for simplified approaches.
Family support	70% (*n* = 14)	“Family members often help older patients remember doses.” (Pharmacist)	Family support: 45.8% pharmacists (Table 5)	Families act as adherence reminders, especially in community settings.
Religious counseling	60% (*n* = 12)	“Framing medicine use as part of caring for the body resonates with some patients.” (Physician)	Religious counseling: 25.6% (Table 5)	Framing adherence as a religious duty can motivate patients.

Themes derived from thematic analysis of 20 interviews (12 pharmacists, 8 physicians). Prevalence reflects percentage of interviewees endorsing each theme. Quotes are illustrative, selected to reflect theme content.

Systemic resource limitations emerged as the most prevalent theme (85% of interviewees), highlighting the strain of high patient volumes and limited access to adherence-monitoring technologies. This aligned with survey findings showing that 68.0% of HCPs reported time constraints and only 12.6% used electronic monitoring devices.

Community pharmacy interactions (65%) underscored the role of frequent patient visits in enabling longer consultations and trust-building, mirroring quantitative evidence that pharmacists, particularly in community settings, spent more time discussing medications than physicians.

Limited training access (65%) and training gaps hindering practice (70%) highlighted the unequal distribution of adherence-related professional development, which corresponded with lower reported use of structured interventions among untrained HCPs.

Patient-centered counseling was emphasized through tailored counseling (70%), where pharmacists described individualized strategies for patients with multimorbidity, aligning with quantitative data showing high rates of patient education among pharmacists (88.3%). Cultural facilitators featured prominently, with prayer as a routine anchor (80%), religious counseling (60%), and family support (70%) illustrating how cultural and familial structures were leveraged to improve adherence, consistent with survey findings on the use of prayer-based reminders and family involvement.

Several patient-level barriers were also highlighted. Cost barriers (70%) were especially common among physicians’ patients, reflecting the survey finding that 57.3% of physicians cited medication costs as a major barrier. Low literacy challenges (65%) and unreliable self-reports (70%) reinforced quantitative evidence of high reliance on self-report measures despite their recognized limitations. Physicians often adopted a clinical outcomes focus (75%)—monitoring laboratory results such as HbA1c—as an indirect measure of adherence.

Themes related to collaboration and care coordination were also prominent. Trust as a motivator (80%) captured the influence of rapport in encouraging adherence, especially in community pharmacy settings. However, fragmented care (75%) and the need for multidisciplinary protocols (65%) reflected persistent barriers to interdisciplinary collaboration, echoing survey data showing only 22.3% of HCPs engaged in regular collaborative practices. Multimorbidity management (80%) illustrated the complexity of coordinating multiple medications for patients with chronic conditions and reinforced the importance of simplified regimens.

Overall, these qualitative themes provide rich context for the quantitative findings, highlighting both the strengths of current adherence practices—such as pharmacist-led education, cultural alignment, and trust-building—and the persistent systemic, educational, and structural challenges that must be addressed to optimize medication adherence in multimorbidity care.

## 4 Discussion

Our mixed-methods study provides a comprehensive overview of how pharmacists and physicians in Jordan report managing medication adherence, revealing both alignment with global best practices and distinctive context-specific challenges. Pharmacists more frequently reported employing patient education and medication regimen simplification as adherence-support strategies, whereas physicians were less likely to report routine use of these approaches. This emphasis on education and simplifying therapy among pharmacists is consistent with global evidence that pharmacist-led counseling and regimen reviews are associated with improved adherence (18–20) and is consistent with findings from Saudi Arabia, where pharmacists frequently delivered patient education and made regimen adjustments as part of adherence support (11). However, gaps in structured counseling remain, as shown in other therapeutic areas in Jordan, where nearly one-quarter of community pharmacists did not review instructions with patients when dispensing proton pump inhibitors (21). Our findings suggest that front-line pharmacists in Jordan tend to leverage these evidence-based strategies. In contrast, physicians in this study appeared to integrate adherence support less systematically, potentially related to heavier clinic workloads and a traditional focus on prioritizing clinical outcome monitoring, a division of focus also seen in other Gulf countries where physicians rely heavily on biomarker-based evaluation of adherence (22).

Another notable finding was the low reported use of objective adherence monitoring tools, with a predominant reliance on self-reports and, less commonly, pill counts. This mirrors patterns observed globally, where self-report questionnaires and patient interviews are among the most frequently used adherence assessment methods in routine care (23, 24). In Saudi Arabia, for example, physicians and pharmacists also reported using subjective methods such as direct inquiry and clinical outcomes over structured tools like the Morisky scale, which were rarely used (25). This preference for subjective methods mat reflect similar constraints across the Middle East—including cost, limited access to electronic monitoring, and lack of integration between pharmacy and clinic records.

Several barriers to adherence management identified in our study, notably time constraints during consultations and patients’ limited health literacy, resonate strongly with the existing literature. Over two-thirds of our respondents reported that busy clinic schedules and heavy workloads left little time for in-depth adherence counseling or follow-up, a barrier echoed in the Saudi study, where time constraints and systemic overload were the most frequently reported challenges by healthcare professionals managing chronic disease adherence (25). In a related context, Alosaimi et al. (26) found that heavy clinical workloads and insufficient consultation time were significant obstacles to effective patient counseling in Saudi tertiary hospitals. Furthermore, such time limitations are well-documented globally; healthcare providers in many countries cite insufficient consultation time as a major impediment to addressing medication adherence (27, 28). Our findings echo those from Europe, where educators noted that “time constraints were highlighted as a significant challenge” in teaching and practicing medication adherence support. Addressing this issue may require system-level changes, such as longer appointment slots for chronic disease management or involvement of additional support staff (e.g., clinical pharmacists or nurses dedicated to adherence follow-up). Additionally, patients’ low health literacy and medication knowledge emerged as a pervasive barrier in the Jordanian context, according to our participants. This too is in line with a regional study conducted in Lebanon where patients with “little drug knowledge” were significantly more likely to be non-adherent (29). Limited understanding of medications—whether due to low general literacy or inadequate counseling—can contribute to misconceptions about side effects or the necessity of therapy that may undermine adherence. In our study, providers observed patients struggling to comprehend instructions or the importance of long-term therapy, which is consistent with evidence that higher health literacy is associated with better adherence (30). Simple interventions like using plain language, visual aids, or translated materials can empower patients with diverse literacy levels. Indeed, our quantitative results indicated that higher patient health literacy was correlated with better adherence, suggesting that efforts to improve patients’ understanding of their treatment may be associated with better adherence outcomes.

On a positive note, we identified important facilitators of adherence that are deeply rooted in the cultural and social fabric of Jordanian society. Many pharmacists and physicians highlighted the role of family support and religious practices in promoting medication-taking. Family involvement—such as spouses or children helping to remind or physically administer medications—was frequently mentioned in relation to higher reported adherence, and this is strongly supported by the literature. Social support is a well-known predictor of adherence success; prior research (including Middle Eastern studies) has shown that patients with strong family or social support report higher rates of taking medications as prescribed (31). Culturally, Jordan’s family structures often involve close-knit households and caregiving by relatives, which our findings suggest may be associated with higher adherence—an insight that aligns with global evidence that enlisting family members is associated with stronger patients’ medication routines (32).

Perhaps the most notable facilitator reported was the use of prayer times as medication reminders. Jordan’s predominantly Muslim population observes five daily prayers at prescribed times, and some providers noted that patients synchronize medication doses with these routine prayer times. “I take my medicine after each prayer. It helps me remember,” as one patient insight from our recent study (33). This culturally tailored strategy, while not widely documented in literature, aligns conceptually with habit-formation techniques in adherence promotion. By tying pill-taking to an existing daily ritual, patients may establish a behavioral cue. Indeed, recent work on habit-building for adherence supports the idea of anchoring medications to regular activities (34). Our finding is among the first to highlight prayer-based reminders in a Middle Eastern cohort; as formal research on this specific tactic is scant, it represents a potential novel contribution of our study. Moreover, it illustrates how religious coping mechanisms may intersect with health behavior—a theme noted elsewhere, such as a systematic review by Elhag et al. (35) which found that spirituality and religiosity were often associated with better adherence among cardiovascular patients. In practice, encouraging patients to link medication schedules with daily prayers or other routine activities (mealtimes, etc.) could be a culturally sensitive adherence tool in Islamic communities. Similarly, recognizing and involving family caregivers as partners in adherence strategies could amplify the support patients receive at home.

Despite these facilitators, our study brought to light a notable gap in interprofessional collaboration. Pharmacists and physicians in Jordan reported only limited coordination in managing medication adherence. Collaboration between healthcare providers is widely regarded as essential for addressing adherence in a holistic way. Prior reviews have emphasized that no single profession can shoulder this task alone—for instance, a Cochrane review concluded that counseling to improve adherence “should be a shared task between nurses, pharmacists and physicians” (36). Furthermore, effective interprofessional collaboration has been linked to improved patient outcomes and reduced healthcare costs. In our regional context, however, the roles of pharmacists and physicians often remain siloed. As noted earlier, studies in the Middle East have observed pharmacists mainly in dispensing roles with minimal direct engagement in therapy management (12). Physicians, on the other hand, may not routinely involve pharmacists in adherence planning or follow-up. This fragmentation may be associated with inconsistent messages or missed opportunities to reinforce adherence. It also places a high burden on physicians to manage adherence on top of acute clinical issues, which, given time constraints, may be related to gaps in adherence support. By contrast, international models show that closer pharmacist-physician collaboration, through collaborative practice agreements or multidisciplinary clinics is associated with more appropriate medication use and improved adherence (37). A qualitative study in Qatar, for instance, highlighted the need for “concerted multidimensional efforts” and specifically recommended expanding pharmacists’ roles in primary care (providing medication reviews, individualized counseling) as a potential approach associated with addressing adherence barriers in diabetes care (38). Our results strongly support such recommendations. Bridging the collaboration gap in Jordan will likely require policy changes and cultural shifts within the health system—including clear delineation of pharmacists’ clinical roles, improved communication channels, and perhaps joint training of providers to foster mutual trust and teamwork. Notably, the high reported impact of any prior training or exposure to collaborative adherence programs among our respondents suggests that when given the tools and opportunities, providers appreciate and capitalize on teamwork to support their patients.

Finally, an important cross-cutting theme is the evident training gap in medication adherence management. Most pharmacists and physicians in our survey acknowledged they never had any formal training on how to assess or improve patient adherence, although most agreed it is a critical part of patient care. This gap also highlighted by Khawagi et al. (25), who reported that most Saudi HCPs had not received structured adherence training, despite acknowledging its importance. Both studies point to an urgent regional need for formal education in adherence promotion across disciplines. This finding also highlighted in a recent survey of 114 universities across Europe found a “lack of consistent teaching on medication adherence” in medical, pharmacy, and nursing curricula, with over 70% of faculty agreeing that adherence education needs to be enhanced (39). Respondents in that study lamented the paucity of guidance and standardization in teaching adherence, which was associated with variable coverage of the topic. Even at the postgraduate level, many clinicians lack awareness of the magnitude of non-adherence and knowledge of effective interventions. In our context, this is reflected in pharmacists and physicians largely learning to handle adherence through on-the-job experience rather than systematic training. Encouragingly, those who had received training (for example, a workshop on motivational interviewing or a course on chronic disease counseling) reported a notably positive association with their practice. This aligns with the notion that targeted education can enhance providers’ skills and confidence in managing adherence (40). It also echoes calls from international bodies to integrate adherence management into health professionals’ training and continuing education. As a novel contribution, our study quantifies this training gap in the Middle Eastern context and underscores the value of filling it. We identified a clear appetite among providers for more knowledge and tools—a finding that should urge educators and professional associations in Jordan to develop dedicated training modules, guidelines, or mentorship programs around medication adherence. In the interim, sharing best-practice resources (such as simple adherence assessment checklists, patient communication techniques, or culturally tailored counseling tips) could empower current practitioners.

Taken together, these findings carry several practical implications. For clinicians, a key message is to prioritize simple, patient-centered adherence interventions in daily practice. Pharmacists should continue to utilize education and regimen simplification. For example, deprescribing unnecessary medications or aligning dosing schedules with patients’ daily routines as these may represent feasible and effective strategies. Physicians, meanwhile, might adopt a more proactive stance by routinely inquiring about adherence (in a blame-free, empathetic manner) and collaborating with pharmacists to address any issues. Both groups can leverage the unique cultural facilitators identified of encouraging patients to link doses with regular daily events (such as prayer times or meals) and involving family members or caregivers as allies in the treatment plan. Simple actions like asking a patient who at home can help them remember medications, or suggesting they use the call to prayer as a cue, cost little but align the care plan with the patient’s environment and values.

For healthcare administrators and policymakers in Jordan and similar settings, the study highlights several areas for improvement. First, strengthening interprofessional collaboration should be a priority. This could involve establishing formal mechanisms for pharmacist-physician communication. For instance, medication adherence clinics or rounds where pharmacists provide input on patients’ medication-taking, or electronic health record systems that allow pharmacists to document adherence-related observations accessible to physicians. Policymakers might consider expanding the role of pharmacists through collaborative practice agreements that permit them to follow up with patients between doctor visits, adjust therapy for adherence simplification, or provide refill counseling without a new prescription at each interval. International precedents show that when pharmacists are empowered and reimbursed for such services, adherence and health outcomes tend to improve (41, 42). Second, addressing time and resource constraints in clinics is crucial. Incorporating adherence assessment as a routine “vital sign” during visits could prompt clinicians to allocate a few minutes to this issue. Alternatively, task-shifting some adherence support to trained nurses, community health workers, or pharmacy staff may be associated with reduced pressure on physicians. For example, a nurse-led follow-up call to patients a week after a clinic visit may be associated with reinforced adherence messages and identification of early difficulties. Our findings also support making adherence counseling a reimbursable service or a quality indicator in healthcare settings. Third, investing in training and education is perhaps the most foundational implication. Professional bodies and healthcare institutions in Jordan should develop continuing education programs focused on medication adherence strategies, communication skills (like motivational interviewing), and use of available adherence aids. On a broader scale, integrating medication adherence topics into medical and pharmacy school curricula may help produce future clinicians better equipped to manage chronic therapies. Given the high impact reported by those who had training in our study, these educational efforts may be linked to better patient outcomes.

The present study also builds directly on our recently published work examining medication adherence among Jordanian patients with multimorbidity (33). While that study provided patient-centered insights into adherence behaviors, barriers, and facilitators, the current research complements it by focusing on the perspectives and practices of healthcare professionals responsible for supporting adherence. Notably, both studies identified economic barriers, limited health literacy, and the value of cultural facilitators such as prayer-based reminders and family involvement as factors associated with adherence. However, the present study adds depth by revealing how pharmacists and physicians operationalize adherence support, the systemic and interprofessional challenges they face, and the association of training gaps with clinical practice. Together, these two investigations offer a comprehensive, multi-stakeholder understanding of adherence management in Jordan, providing a stronger evidence base for developing integrated, culturally tailored, and system-level interventions to improve long-term medication adherence.

## 5 Conclusion

This mixed-methods study provides a comprehensive understanding of how pharmacists and physicians in Jordan manage medication adherence, highlighting both strengths and areas for improvement. Pharmacists demonstrated a greater emphasis on patient education and regimen simplification, while physicians tended to focus on clinical outcome monitoring. Barriers such as time constraints, low patient health literacy, and limited interprofessional collaboration persist, yet culturally embedded facilitators—particularly prayer-based reminders and family support—offer valuable opportunities for tailored interventions. The findings highlight the crucial need for structured adherence training, system-level strategies to alleviate workload pressures, and stronger multidisciplinary collaboration to optimize patient support. Leveraging cultural facilitators alongside evidence-based practices can enhance adherence management in Jordan and inform similar strategies across the Middle East.

## 6 Limitations

This study should be interpreted in light of several limitations. First, the use of a cross-sectional design limits the ability to establish causal relationships between healthcare professionals’ practices and patient adherence outcomes. Second, although a mixed-methods approach provided a comprehensive view of adherence management, the quantitative data relied on self-reported responses, which may be subject to recall bias and social desirability bias. This reliance may have led participants to overestimate their engagement in adherence-support strategies or underestimate barriers, potentially affecting the accuracy of reported prevalence rates. Third, the survey used convenience sampling for the quantitative phase. While participants were recruited from a variety of regions, practice settings, and professional backgrounds, the resulting sample does not fully mirror the national distribution of healthcare professionals in Jordan. This introduces the possibility of selection bias and limits the representativeness of findings. Consequently, the prevalence of reported practices and barriers should be interpreted with caution, as they may not fully reflect the broader population of healthcare professionals in Jordan. Future studies could enhance representativeness by using probability-based sampling strategies, such as stratified random sampling from professional licensing registers, or multi-stage cluster sampling that includes rural and underserved regions. Such approaches would allow more robust generalization of findings to the national HCP population. The qualitative phase purposively included participants with varied backgrounds to enhance diversity, but the relatively small number of interviewees (*n* = 20) means some perspectives may not be fully captured. Fourth, the study focused exclusively on pharmacists and physicians; other healthcare providers such as nurses, dietitians, and community health workers, who may also play important roles in adherence promotion, were not included.

## Data Availability

The original contributions presented in the study are included in the article/supplementary material, further inquiries can be directed to the corresponding author.

## References

[1] Aremu TO Oluwole OE Adeyinka KO Schommer JC. Medication adherence and compliance: recipe for improving patient outcomes. Pharmacy. (2022) 10. doi: 10.3390/pharmacy10050106, PMID: 36136839 PMC9498383

[2] Religioni U Barrios-Rodríguez R Requena P Borowska M Ostrowski J. Enhancing therapy adherence: impact on clinical outcomes, healthcare costs, and patient quality of life. Medicina (Kaunas). (2025) 61. doi: 10.3390/medicina61010153, PMID: 39859135 PMC11766829

[3] Eduardo S. Adherence to long-term therapies: evidence for action. Geneva: World Health Organization (2017).

[4] Kvarnström K Westerholm A Airaksinen M Liira H. Factors contributing to medication adherence in patients with a chronic condition: a scoping review of qualitative research. Pharmaceutics. (2021) 13. doi: 10.3390/pharmaceutics13071100, PMID: 34371791 PMC8309154

[5] Barakat M Nassar R Gharaibeh L Thiab S Nashwan AJ. Current landscape and future directions of Deprescribing and polypharmacy practices in Jordan. Med Princ Pract Int J Kuwait Univ Heal Sci Cent. (2024) 33:1–14. doi: 10.1159/000541009, PMID: 39159605 PMC11631112

[6] Fiorillo A Barlati S Bellomo A Corrivetti G Nicolò G Sampogna G . The role of shared decision-making in improving adherence to pharmacological treatments in patients with schizophrenia: a clinical review. Ann General Psychiatry. (2020) 19:43. doi: 10.1186/s12991-020-00293-4, PMID: 32774442 PMC7409631

[7] Marcum ZA Jiang S Bacci JL Ruppar TM. Pharmacist-led interventions to improve medication adherence in older adults: a meta-analysis. J Am Geriatr Soc. (2021) 69:3301–11. doi: 10.1111/jgs.17373, PMID: 34287846 PMC8595553

[8] Brown MT Bussell JK. Medication adherence: WHO cares? Mayo Clin Proc. (2011) 86:304–14. doi: 10.4065/mcp.2010.0575, PMID: 21389250 PMC3068890

[9] Hatah E Lim KP Ali AM Mohamed Shah N Islahudin F. The influence of cultural and religious orientations on social support and its potential impact on medication adherence. Patient Prefer Adherence. (2015) 9:589–96. doi: 10.2147/PPA.S79477, PMID: 25960641 PMC4423506

[10] Peh KQE Kwan YH Goh H Ramchandani H Phang JK Lim ZY . An adaptable framework for factors contributing to medication adherence: results from a systematic review of 102 conceptual frameworks. J Gen Intern Med. (2021) 36:2784–95. doi: 10.1007/s11606-021-06648-1, PMID: 33660211 PMC8390603

[11] Alhomoud FK Alwohaibi LW Aljarrash K Alhomoud F Alamer K Alsultan MM . Evaluating strategies for enhancing medication adherence in the Kingdom of Saudi Arabia (KSA): a cross-sectional study. Patient Prefer Adherence. (2024) 18:2469–80. doi: 10.2147/PPA.S499795, PMID: 39669314 PMC11636242

[12] Alsairafi Z Waheedi M Alsaleh F. The perspectives of patients and physicians on the role of pharmacists in improving medication adherence in type 2 diabetes: a qualitative study. Patient Prefer Adherence. (2019) 13:1527–43. doi: 10.2147/PPA.S218068, PMID: 31571836 PMC6750862

[13] Clyne W Mshelia C Hall S McLachlan S Jones P Dobbels F . Management of patient adherence to medications: protocol for an online survey of doctors, pharmacists and nurses in Europe. BMJ Open. (2011) 1:e000355. doi: 10.1136/bmjopen-2011-000355, PMID: 22080529 PMC3276023

[14] Kamusheva M Aarnio E Qvarnström M Hafez G Mucherino S Potočnjak I . Pan-European survey on medication adherence management by healthcare professionals. Br J Clin Pharmacol. (2024) 90:3135–45. doi: 10.1111/bcp.16183 39073168 PMC11602874

[15] Kvarnström K Airaksinen M Liira H. Barriers and facilitators to medication adherence: a qualitative study with general practitioners. BMJ Open. (2018) 8:e015332. doi: 10.1136/bmjopen-2016-015332, PMID: 29362241 PMC5786122

[16] Molitorisová M Kotlářová J Snopkova M Waczulikova I. Support of medication adherence by community pharmacists in Czech and Slovak republics: a questionnaire survey study. Eur Pharm J. (2017) 65:15–23. doi: 10.1515/afpuc-2017-0006

[17] Peterson AM Takiya L Finley R. Meta-analysis of trials of interventions to improve medication adherence. Am J Heal Pharm AJHP Off J Am Soc Heal Pharm. (2003) 60:657–65. doi: 10.1093/ajhp/60.7.657, PMID: 12701547

[18] Cutler RL Fernandez-Llimos F Frommer M Benrimoj C Garcia-Cardenas V. Economic impact of medication non-adherence by disease groups: a systematic review. BMJ Open. (2018) 8:e016982. doi: 10.1136/bmjopen-2017-016982, PMID: 29358417 PMC5780689

[19] Farhana L Rahayu FP Sholihah S Sweileh W Abdulah R Alfian SD. Effectiveness of pharmacist-led intervention on medication adherence in chronic diseases: a systematic review of randomized controlled trials. Patient Prefer Adherence. (2025) 19:2161–78. doi: 10.2147/PPA.S530503 40726768 PMC12301242

[20] Viswanathan M Kahwati LC Golin CE Blalock SJ Coker-Schwimmer E Posey R . Medication therapy management interventions in outpatient settings: a systematic review and meta-analysis. JAMA Intern Med. (2015) 175:76–87. doi: 10.1001/jamainternmed.2014.5841, PMID: 25401788

[21] Gharaibeh L Alameri MA Al-Hawamdeh MI Daoud E Atwan R Lafi Z . Practices and knowledge of community pharmacists towards the use of proton pump inhibitors: a cross-sectional study in Jordan. BMJ Open. (2025) 15:e085589. doi: 10.1136/bmjopen-2024-085589, PMID: 39922594 PMC11808909

[22] Alhaddad IA Hamoui O Hammoudeh A Mallat S. Treatment adherence and quality of life in patients on antihypertensive medications in a middle eastern population: adherence. Vasc Health Risk Manag. (2016) 12:407–13. doi: 10.2147/VHRM.S105921, PMID: 27822055 PMC5089866

[23] Lam WY Fresco P. Medication adherence measures: an overview. Biomed Res Int. (2015) 2015:217047. doi: 10.1155/2015/217047, PMID: 26539470 PMC4619779

[24] Saha SK Adhikary A Jha A Mehta V. Use of interventions to overcome medication non-adherence. Int J Asian Bus Inf Manag. (2021) 12:289–318. doi: 10.4018/IJABIM.20210701.oa18, PMID: 41380940

[25] Khawagi WY Bansal N Shang N Chen L-C. A systematic review of potential opioid prescribing safety indicators. Pharmacoepidemiol Drug Saf. (2025) 4. doi: 10.3390/pharma4010004

[26] Alosaimi K Alwafi H Alhindi Y Falemban A Alshanberi A Ayoub N . Medication adherence among patients with chronic diseases in Saudi Arabia. Int J Environ Res Public Health. (2022) 19. doi: 10.3390/ijerph191610053, PMID: 36011690 PMC9408114

[27] Kerr D Ostaszkiewicz J Dunning T Martin P. The effectiveness of training interventions on nurses’ communication skills: a systematic review. Nurse Educ Today. (2020) 89:104405. doi: 10.1016/j.nedt.2020.104405, PMID: 32244125

[28] Nagel S Schlesinger T Bayle E Giauque D. Professionalisation of sport federations – a multi-level framework for analysing forms, causes and consequences. Eur Sport Manag Q. (2015) 15:407–33. doi: 10.1080/16184742.2015.1062990

[29] Al-Hajje A Awada S Rachidi S Zein S Bawab W El-Hajj Z . Factors affecting medication adherence in Lebanese patients with chronic diseases. Pharm Pract (Granada). (2015) 13:590. doi: 10.18549/PharmPract.2015.03.590, PMID: 26445621 PMC4582745

[30] Miller TA. Health literacy and adherence to medical treatment in chronic and acute illness: a meta-analysis. Patient Educ Couns. (2016) 99:1079–86. doi: 10.1016/j.pec.2016.01.020, PMID: 26899632 PMC4912447

[31] Shahin W Kennedy GA Stupans I. The association between social support and medication adherence in patients with hypertension: a systematic review. Pharm Pract (Granada). (2021) 19:2300. doi: 10.18549/PharmPract.2021.2.2300, PMID: 34221197 PMC8234709

[32] DiMatteo MR. Social support and patient adherence to medical treatment: a meta-analysis. Health Psychol. (2004) 23:207–18. doi: 10.1037/0278-6133.23.2.207, PMID: 15008666

[33] Abed A Abu Assab M Abu Dayyih W Alotaibi BS Alsubaie N. Medication adherence in Jordanian patients with multimorbidity: a cross-sectional mixed-methods study in outpatient clinics. Front Pharmacol. (2025) 16. doi: 10.3389/fphar.2025.1619023, PMID: 40822490 PMC12351122

[34] Badawy SM Shah R Beg U Heneghan MB. Habit strength, medication adherence, and habit-based Mobile health interventions across chronic medical conditions: systematic review. J Med Internet Res. (2020) 22:e17883. doi: 10.2196/17883, PMID: 32343250 PMC7218590

[35] Elhag M Awaisu A Koenig HG Mohamed Ibrahim MI. The association between religiosity, spirituality, and medication adherence among patients with cardiovascular diseases: a systematic review of the literature. J Relig Health. (2022) 61:3988–4027. doi: 10.1007/s10943-022-01525-5, PMID: 35274225 PMC9509306

[36] Nieuwlaat R Wilczynski N Navarro T Hobson N Jeffery R Keepanasseril A . Interventions for enhancing medication adherence. Cochrane Database Syst Rev. (2014) 2014:CD000011. doi: 10.1002/14651858.CD000011.pub4, PMID: 25412402 PMC7263418

[37] Pellegrino AN Martin MT Tilton JJ Touchette DR. Medication therapy management services: definitions and outcomes. Drugs. (2009) 69:393–406. doi: 10.2165/00003495-200969040-00001, PMID: 19323584

[38] Jaam M Mohamed Ibrahim MI Kheir N Hadi MA Diab MI Awaisu A. Assessing prevalence of and barriers to medication adherence in patients with uncontrolled diabetes attending primary healthcare clinics in Qatar. Prim Care Diabetes. (2018) 12:116–25. doi: 10.1016/j.pcd.2017.11.001, PMID: 29170095

[39] Gottlieb H Seghers L Leiva-Fernandez F Ghiciuc CM Hafez G Herdeiro MT . Medication adherence in the curricula of future European physicians, pharmacists and nurses – a cross-sectional survey. BMC Med Educ. (2025) 25:339. doi: 10.1186/s12909-025-06909-1 40045247 PMC11881433

[40] Marie-Schneider P Aslani P. Adherence policy, education and practice - an international perspective. Pharm Pract (Granada). (2010) 8:209–12. doi: 10.4321/s1886-36552010000400001 25126142 PMC4127057

[41] Carter BL Coffey CS Ardery G Uribe L Ecklund D James P . Cluster-randomized trial of a physician/pharmacist collaborative model to improve blood pressure control. Circ Cardiovasc Qual Outcomes. (2015) 8:235–43. doi: 10.1161/CIRCOUTCOMES.114.001283, PMID: 25805647 PMC4618490

[42] Farooqui M Alharbi H Alamro A Almoshawe M Suhaj A Ahmad R. Role of pharmacists in clinical pharmacy services in Saudi Arabia: a survey of knowledge, attitude, and practice. J Hunan Univ Nat Sci. (2023) 31. doi: 10.55463/issn.1674-2974.50.9.4

